# Comparison of triglyceride glucose index, and related parameters to predict insulin resistance in Korean adults: An analysis of the 2007-2010 Korean National Health and Nutrition Examination Survey

**DOI:** 10.1371/journal.pone.0212963

**Published:** 2019-03-07

**Authors:** Jinsook Lim, Jimyung Kim, Sun Hoe Koo, Gye Cheol Kwon

**Affiliations:** Department of Laboratory Medicine, Chungnam National University Hospital, Daejeon, South Korea; Mexican Social Security Institute, MEXICO

## Abstract

The triglyceride glucose (TyG) index, a product of triglyceride and fasting glucose, is a reliable marker for insulin resistance (IR). Obesity is also known to be closely related with IR. Recently, the efficiency of TyG-related markers that combine obesity markers with TyG index has been studied; however, earlier studies were limited in number and the results were inconsistent. Therefore, in this study, we investigated the efficiency of several combinations of TyG index and obesity indices, namely, body mass index (BMI), waist circumference (WC), and waist-to-height ratio (WHtR), in reflecting IR. Data were obtained from the Korean National Health and Nutrition Examination Survey from 2007–2010. A total of 11,149 subjects (4,777 men and 6,372 women) were included. IR was defined as the homeostasis model assessment for IR (HOMA-IR) of above the 75^th^ percentile for each gender. Logistic regression analysis was performed after adjusting for confounding factors, to compare and identify the associations of the 4 parameters (TyG index, TyG-BMI, TyG-WC, and TyG-WHtR) with IR. For each parameter, odds ratios (OR) and 95% confidence intervals (CIs) of quartiles 2–4 were calculated and compared with quartile 1 as a reference. A receiver operating characteristic (ROC) curve analysis was conducted to evaluate the ability of each parameter to predict IR. The adjusted ORs of quartile 4 in comparison with quartile 1 (95% CIs) for IR were 7.60 (6.52–8.87) for TyG index, 12.82 (10.89–15.10) for TyG-BMI, 16.29 (13.70–19.38) for TyG-WC, and 14.86 (12.53–17.62) for TyG-WHtR. The areas under the ROC curve for each parameter were 0.690 for TyG index, 0.748 for TyG-BMI, 0.731 for TyG-WC, and 0.733 for TyG-WHtR. In conclusion, TyG-BMI was found to be superior to other parameters for IR prediction. We propose TyG-BMI as an alternative marker for assessing IR in clinical settings.

## Introduction

Insulin resistance (IR) is characterized by an inappropriate physiologic response in which insensitivity to insulin results in compensatory hyperinsulinemia [1]. IR is known to be a major risk factor for metabolic syndrome, type 2 diabetes and cardiovascular diseases [2–4]. Rapidly growing population with chronic diseases associated with IR are reported worldwide, including among the Korean population [5,6]. Thus, early detection of IR is crucial to prevent the manifestation of clinical diseases. The glucose clamp technique, first described by DeFronzo is considered as the gold standard for quantification of IR [7]. However, it is difficult to perform in routine laboratory because of its complexity and invasiveness [8]. To overcome these problems, the homeostasis model for IR (HOMA-IR) was developed in 1985 [9] and has been widely used for IR quantification. However, insulin measurement is still not readily available in many routine laboratories and entails standardization issues [10]. Consequently, several studies have explored methods that can easily predict IR in routine laboratory assessments and they proposed various markers such as lipid ratios and visceral adiposity index (VAI) [8,11–16]. The triglyceride glucose (TyG) index, a product of fasting triglycerides and glucose, has also recently been suggested for IR estimation, and this measurement demonstrates improved efficiency compared to previous markers [8,17,18].

In addition to TyG index, obesity has been shown to be associated with IR. Obesity indices, namely, body mass index (BMI), waist circumference (WC), and waist-to-height ratio (WHtR) have been widely used because of their easy practical application. Several studies have evaluated TyG-related parameters that combined obesity indices and TyG index for IR or diabetes, such as TyG-BMI or TyG-WC, and found that they are more efficient than TyG index alone [19,20]; however, a major limitation of these studies is the small number of participants. Thus, additional studies are needed to compare these IR markers. In addition, no studies have yet been conducted using TyG index combined with WHtR. Therefore, in this study, we compared TyG index and TyG-related parameters (TyG-WC, TyG-BMI and TyG-WHtR) for the detection of IR.

## Materials and methods

### Study population

Data were obtained from the Korean National Health and Nutrition Examination Survey (KNHANES) covering the period 2007–2010. The KNHANES is an annual cross-sectional survey conducted by the Korean Ministry of Health and Welfare. All participants were randomly selected, voluntarily enrolled in the survey and provided an informed consent. This survey was approved by the institutional review board of the Korea Centers for Disease Control (KCDC) [21]. The subjects of the present study were adults aged 20 years or older. Subjects with chronic liver or kidney disease, any type of cancer, a history of myocardial infarction, or stroke were excluded. Participants diagnosed with diabetes, hypertension, or hyperlipidemia and those taking regular medication were also excluded, as were participants with missing data (demographic, anthropometric, or laboratory), those who did not fast for at least 8 hours before testing and those with extreme BMI (≥ 40 kg/m^2^), TG (>500 mg/dL) or HDL (>100 mg/dL). Finally, a total of 11,149 subjects (4,777 men and 6,372 women) were included in our analysis.

### Anthropometric measurements

Physical examinations were performed by trained staffs according to a standardized protocol. Body weight and height were measured with the subject wearing light indoor clothing and BMI was calculated using the formula: BMI = weight (kg) / height (m^2^). WC was measured midway between the costal margin and the iliac crest at the end of a normal expiration, and WHtR was calculated using the formula: WHtR = WC (cm)/ height (cm). Blood pressure (BP) was measured three times and the average values for systolic and diastolic BPs were used for analysis.

### Laboratory measurements and calculations

Blood samples were obtained after at least an 8-hour overnight fast. The venous blood sample was then delivered to the central laboratory. Levels of fasting blood glucose, triglycerides, total cholesterol, and high-density lipoprotein cholesterol (HDL-C) were measured using an ADVIA 1650 chemistry analyzer (Siemens, Washington, DC, USA) in 2007, and a Hitachi 7600 automatic analyzer (Hitachi, Tokyo, Japan) from 2008 to 2010. For HDL-C, corrected values using the conversion equations recommended by the KCDC were used [22]. LDL-C was calculated using the Friedewald equation. Serum insulin was measured by 1470 Wizard gamma counter (Perkin-Elmer, Turku, Finland).

For quantification of IR, HOMA-IR was calculated as follows: HOMA-IR = fasting insulin (μU/dL) × fasting glucose (mg/dL) / 22.5. TyG index, TyG-WC, TyG-BMI were calculated using the formula published previously. The TyG index [17,18]: Ln[TG (mg/ dL) × fasting glucose (mg/dL)/2]. TyG-BMI, TyG-WC and TyG-WHtR indicate TyG index x BMI [20], TyG index x WC [20] and TyG index x WHtR, respectively.

### Classification of variables

IR was defined as the homeostasis model assessment for IR (HOMA-IR) of above the 75^th^ percentile for each gender [23,24]. Self-reported questionnaires were used to determine smoking status, alcohol consumption, and exercise habits. Current smoking was defined as subjects who were currently smoking and had smoked more than 100 cigarettes in their lifetime. Alcohol drinking was defined as drinking at least twice a week during a year. Regular exercise was defined as exercising with moderate to vigorous intensity at least three days per week.

### Statistical analysis

All data were analyzed using SPSS version 20.0 for Windows (SPSS, Chicago, IL, USA) and MedCalc Statistical Software version 18.6 (MedCalc Software bvba, Ostend, Belgium). The data are presented as mean ± standard deviation (SD) for continuous variables, and as percentage for categorical variables. The independent sample t-test was used to compare continuous variables and the Chi-square test was used to compare categorical variables. For odds ratios (ORs) and 95% confidence intervals (CIs) of various markers for IR, the stepwise logistic regression analyses were performed to examine the relation between IR as the dependent variable and various markers, by controlling confounding factors (age, gender, systolic pressure, diastolic pressure, smoking, drinking, and exercise). ORs and 95% CIs of quartiles 2–4 for each parameter were calculated and compared with quartile 1 as a reference. Receiver operating characteristic (ROC) curves were plotted and the area under the curve (AUC) was calculated to compare the relative diagnostic strengths of these parameters for identifying IR. Pairwise comparisons between AUCs for the four parameters were performed according to DeLong et al [25]. All statistical tests were two-sided, and a value of P *<* 0.05 was considered statistically significant.

## Results

A total of 11,149 participants were included in the study, including 8,362 without IR (Non-IR group) and 2,787 with IR (IR group). The anthropometric, biochemical, and clinical characteristics of participants by gender and presence of IR are summarized in Table 1. The mean ages of the non-IR and IR groups were 44.7 ± 14.9 and 44.5 ± 14.3 years, respectively. Age, height, smoking, drinking, and regular exercise were not significantly different between non-IR and IR groups. However, mean body weight, waist circumference, BMI, WHtR, systolic and diastolic BPs, glucose, total cholesterol, TG, LDL, insulin, HOMA-IR, TyG index, TyG-BMI, TyG-WC, and TyG-WHtR, were significantly higher while the mean HDL level was significantly lower in the IR group than in the non-IR group.

**Table 1:** Baseline characteristics of participants according to gender and presence of insulin resistance.

	Male (n = 4777)	Female (n = 6372)	P-value	Non-IR (n = 8362)	IR (n = 2787)	P-value
Age (years)	45.2 ± 15.0	44.3 ± 14.6	0.001	44.7 ± 14.9	44.5 ± 14.3	0.457
Height (cm)	170.4 ± 6.6	157.5 ± 6.4	<0.001	162.9 ± 9.0	163.3 ± 9.3	0.065
Weight (kg)	69.2 ± 10.8	56.7 ± 8.8	<0.001	60.2 ± 10.5	67.6 ± 12.7	<0.001
Waist circumference (cm)	83.5 ± 8.6	76.9 ± 9.3	<0.001	77.9 ± 8.8	85.0 ± 9.9	<0.001
BMI (kg/m^2^)	23.8 ± 3.1	22.9 ± 3.3	<0.001	22.6 ± 2.9	25.2 ± 3.5	<0.001
WHtR	0.49 ± 0.05	0.49 ± 0.06	0.418	0.48 ± 0.05	0.52 ± 0.06	<0.001
Smoking, n (%)	1450 (30.4%)	249 (3.9%)	<0.001	1278 (15.3%)	421 (15.1%)	0.821
Drinking, n (%)	1799 (37.7%)	615 (9.7%)	<0.001	1835 (21.9%)	579 (20.8%)	0.194
Regular exercise, n (%)	2714 (56.8%)	2709 (42.5%)	<0.001	4111 (49.2%)	1312 (47.1%)	0.056
Systolic blood pressure (mmHg)	117.9 ± 15.2	111.6 ± 16.3	<0.001	113.2 ± 16.0	117.7 ± 16.2	<0.001
Diastolic blood pressure (mmHg)	77.6 ± 10.4	71.9 ± 9.8	<0.001	73.6 ± 10.3	76.8 ± 10.5	<0.001
Glucose (mg/mL)	94.8 ± 16.8	91.3 ± 13.0	<0.001	89.9 ± 8.8	101.4 ± 23.5	<0.001
Total cholesterol (mg/mL)	188.1 ± 34.6	186.4 ± 35.5	0.016	184.7 ± 34.4	194.5 ± 36.2	<0.001
Triglycerides (mg/mL)	140.8 ± 82.0	103.5 ± 61.7	<0.001	109.8 ± 66.8	148.5 ± 84.3	<0.001
High-density lipoprotein (mg/mL)	46.3 ± 10.2	51.1 ± 10.8	<0.001	50.0 ± 10.9	46.0 ± 10.0	<0.001
Low-density lipoprotein (mg/mL)	114.8 ± 30.2	110.6 ± 31.8	<0.001	112.7 ± 30.6	118.8 ± 33.1	<0.001
Insulin (μIU/mL)	9.62 ± 4.54	9.57 ± 4.19	0.549	7.89 ± 1.88	14.7 ± 5.4	<0.001
HOMA-IR	2.28 ± 1.30	2.19 ± 1.18	<0.001	1.75 ± 0.44	3.66 ± 1.66	<0.001
TyG index	8.64 ± 0.60	8.31 ± 0.57	<0.001	8.35 ± 0.57	8.76 ± 0.60	<0.001
TyG-BMI	206.2 ± 34.3	190.6 ± 34.6	<0.001	189.3 ± 30.6	221.5 ± 37.3	<0.001
TyG-WC	723.4 ± 104.4	641.1 ± 104.1	<0.001	652.8 ± 100.6	746.9 ± 114.4	<0.001
TyG-WHtR	4.25 ± 0.62	4.08 ± 0.70	<0.001	4.01 ± 0.61	4.58 ± 0.68	<0.001
Insulin resistance	1194 (25.0%)	1593 (25.0%)	0.995			

Abbreviations: IR, insulin resistance; BMI, body mass index; WHtR, waist to height ratio; HOMA-IR, the homeostasis model assessment for insulin resistance; TyG index, a product of triglyceride and fasting glucose; WC, waist circumference.

The ORs and 95% CIs for IR were progressively increased across quartiles of each parameter both before and after adjustment (Table 2). TyG-WC presented the highest ORs and 95% CIs for IR, reaching 16.29 (95% CI 13.70–19.38) for the top quartile compared with the bottom quartile (P < 0.001), followed by TyG-WHtR (Q4 14.86, 95% CI 12.53–17.62) and TyG-BMI (Q4 12.82, 95% CI 10.89–15.10).

**Table 2:** Odds ratios and adjusted odds ratios for insulin resistance in quartiles of each parameter.

All subjects	Unadjusted OR (95% CI)	P-value	Adjusted OR* (95% CI)	P-value
TyG index				
Q1	1		1	
Q2	1.85 (1.59–2.15)	<0.001	2.03 (1.74–2.37)	<0.001
Q3	2.93 (2.53–3.39)	<0.001	3.40 (2.92–3.95)	<0.001
Q4	6.14 (5.34–7.06)	<0.001	7.60 (6.52–8.87)	<0.001
TyG-BMI				
Q1	1		1	
Q2	1.83 (1.55–2.16)	<0.001	2.03 (1.71–2.40)	<0.001
Q3	3.46 (2.96–4.04)	<0.001	4.12 (3.50–4.85)	<0.001
Q4	10.53 (9.06–12.23)	<0.001	12.82 (10.89–15.10)	<0.001
TyG-WC				
Q1	1		1	
Q2	1.93 (1.64–2.27)	<0.001	2.39 (2.02–2.83)	<0.001
Q3	3.43 (2.94–4.00)	<0.001	5.05 (4.28–5.96)	<0.001
Q4	9.30 (8.02–10.79)	<0.001	16.29 (13.70–19.38)	<0.001
TyG-WHtR				
Q1	1		1	
Q2	2.09 (1.77–2.47)	<0.001	2.49 (2.10–2.95)	<0.001
Q3	3.62 (3.10–4.24)	<0.001	5.00 (4.23–5.90)	<0.001
Q4	9.90 (8.52–11.52)	<0.001	14.86 (12.53–17.62)	<0.001
**Male**				
TyG index				
Q1	1		1	
Q2	2.09 (1.51–2.90)	<0.001	2.15 (1.55–2.99)	<0.001
Q3	3.86 (2.84–5.23)	<0.001	3.99 (2.93–5.43)	<0.001
Q4	8.19 (6.10–10.98)	<0.001	8.57 (6.33–11.59)	<0.001
TyG-BMI				
Q1	1		1	
Q2	1.95 (1.37–2.78)	<0.001	1.91 (1.34–2.72)	<0.001
Q3	4.01 (2.89–5.56)	<0.001	3.95 (2.84–5.50)	<0.001
Q4	13.74 (10.04–18.80)	<0.001	13.40 (9.72–18.45)	<0.001
TyG-WC				
Q1	1		1	
Q2	2.21 (1.39–3.49)	<0.001	2.21 (1.39–3.51)	<0.001
Q3	4.61 (2.99–7.10)	<0.001	4.89 (3.16–7.56)	<0.001
Q4	16.04 (10.54–24.40)	<0.001	17.18 (11.21–26.33)	<0.001
TyG-WHtR				
Q1	1		1	
Q2	2.61 (1.86–3.67)	<0.001	2.85 (2.02–4.02)	<0.001
Q3	5.49 (3.99–7.55)	<0.001	6.82 (4.91–9.47)	<0.001
Q4	15.95 (11.67–21.81)	<0.001	20.40 (14.69–28.33)	<0.001
**Female**				
TyG index				
Q1	1		1	
Q2	1.92 (1.61–2.28)	<0.001	2.03 (1.70–2.42)	<0.001
Q3	2.96 (2.49–3.52)	<0.001	3.21 (2.68–3.85)	<0.001
Q4	6.79 (5.69–8.10)	<0.001	7.47 (6.16–9.05)	<0.001
TyG-BMI				
Q1	1		1	
Q2	1.94 (1.60–2.35)	<0.001	2.14 (1.76–2.60)	<0.001
Q3	3.80 (3.16–4.56)	<0.001	4.43 (3.65–5.39)	<0.001
Q4	11.33 (9.41–13.63)	<0.001	12.98 (10.59–15.89)	<0.001
TyG-WC				
Q1	1		1	
Q2	2.21 (1.85–2.64)	<0.001	2.50 (2.09–3.01)	<0.001
Q3	4.35 (3.65–5.18)	<0.001	5.36 (4.44–6.48)	<0.001
Q4	12.18 (10.09–14.71)	<0.001	15.69 (12.69–19.40)	<0.001
TyG-WHtR				
Q1	1		1	
Q2	2.10 (1.73–2.55)	<0.001	2.49 (2.05–3.03)	<0.001
Q3	3.32 (2.75–4.01)	<0.001	4.48 (3.66–5.49)	<0.001
Q4	8.56 (7.15–10.26)	<0.001	13.49 (10.91–16.69)	<0.001

Abbreviations: OR, odds ratio; CI, confidence interval; TyG index, a product of triglyceride and fasting glucose; BMI, body mass index; WC, waist circumference; WHtR, waist to height ratio.

*Odds ratios for all subjects were adjusted for age, gender, systolic pressure, diastolic pressure, smoking, drinking, and exercise.

**Fig 1:**
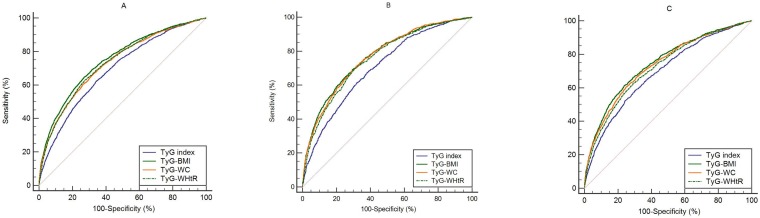
Receiver operating characteristic (ROC) curve of each parameter for predicting insulin resistance. (A) ROC curve for predicting insulin resistance in all subjects. (B) ROC curve for predicting insulin resistance in male. (C) ROC curve for predicting insulin resistance in female. Abbreviations: TyG index, a product of triglyceride and fasting glucose; BMI, body mass index; WC, waist circumference; WHtR, waist-to-height ratio.

The results of ROC curve analyses and AUCs with their corresponding 95% CIs for TyG index, TyG-BMI, TyG-WC, and TyG-WHtR, are shown in Table 3 and Fig 1. TyG-BMI showed the largest AUC for IR detection (0.748, 95% CI 0.740–0.756), followed by TyG-WHtR (0.733, 95% CI 0.725–0.742) and TyG-WC (0.731, 95% CI 0.722–0.739) in all subjects. When analyzed by gender, TyG-BMI showed the largest AUC in both males and females (0.769, 95% CI 0.757–0.781, and 0.745, 95% CI 0.734–0.756, respectively) suggesting that TyG-BMI has the best discriminative power to predict IR when compared to other parameters, in both genders.

**Table 3:** Areas under the receiver operating characteristic curves for each parameter for predicting insulin resistance.

Parameters	Area under the curve	95% CI	P-value
**All subjects**			
TyG index	0.690	0.681–0.698	<0.001
TyG-BMI	0.748	0.740–0.756	<0.001
TyG-WC	0.731*	0.722–0.739	<0.001
TyG-WHtR	0.733	0.725–0.742	<0.001
**Male**			
TyG index	0.704	0.691–0.717	<0.001
TyG-BMI	0.769**	0.757–0.781	<0.001
TyG-WC	0.766	0.754–0.778	<0.001
TyG-WHtR	0.755	0.743–0.767	<0.001
**Female**			
TyG index	0.691	0.680–0.702	<0.001
TyG-BMI	0.745	0.734–0.756	<0.001
TyG-WC	0.735	0.723–0.745	<0.001
TyG-WHtR	0.723	0.712–0.734	<0.001

Abbreviations: CI, confidence interval; TyG index, a product of triglyceride and fasting glucose; BMI, body mass index; WC, waist circumference; WHtR, waist to height ratio.

* P = 0.157 in comparison of TyG-WC and TyG-WHtR

** P = 0.446 in comparison of TyG-BMI and TyG-WC

P<0.001 for all of the other comparisons

## Discussion

In this cross-sectional study, we evaluated and compared four parameters of IR: TyG index, TyG-BMI, TyG-WC, and TyG-WHtR. Overall, the combination of obesity indices with TyG index showed better results than TyG index alone. Moreover, we found that TyG-BMI, a combination of TyG index and BMI, performed better than the other parameters with a higher odds ratio and the largest AUC of 0.748 in all subjects.

IR is known to be the core pathological mechanism for type 2 diabetes, and to precede the diagnosis of type 2 DM [26,27]. Therefore, detection of IR for people at risk is important. There have been many attempts to detect IR at a lower cost and by a simpler method to overcome the practical limitations of the glucose clamp technique, and high cost and unavailability of insulin measurement required for HOMA-IR in routine laboratories [11,13,15,28]. Among these, the TyG index proposed by Guerrero-Romero et al. has shown high sensitivity and specificity in the detection of IR and therefore it was considered the reliable marker for IR in several studies [8,17,18]. IR, by definition, is a state of high insulin levels due to insulin insensitivity, which correlates well with triglycerides levels [29]. Indeed, hypertriglyceridemia may be related to the increased transport of free fatty acids to the liver, resulting in an increase in hepatic glucose output [30]. Therefore, TyG index, a product of triglycerides and glucose, can predict insulin resistance better than other available markers. In addition to TyG index, the association between obesity and IR is also well established. Accordingly, a combination of TyG index and obesity indices can be expected to predict IR better than TyG index alone.

In our study, the performance of TyG index combined with obesity indices was considerably better than the performance of TyG index alone. Of the parameters evaluated, we found that TyG-BMI showed the best discriminative ability. Visceral (intra-abdominal) fat deposits are known to play a more important roles in the development of IR than subcutaneous fat deposits, because they produce more fatty acids and secrete inflammatory cytokines and adipokines [20,30–32]. Accordingly, when WC or WHtR, markers of visceral adiposity, are combined with TyG index, they should be expected to predict IR more successfully than BMI. However, in our study, a combination of TyG index and BMI, a general obesity marker, gave the best predictive value for IR. Abdominal obesity includes both subcutaneous and visceral adipose tissue, of which the latter is known to have more effect on IR [33]. However, WC cannot differentiate subcutaneous and visceral fat, and therefore it cannot fully represent visceral fat [20]. Furthermore, WC does not reflect the effect of height on cardiometabolic risk [34,35], causing its efficacy declines for individuals who are tall or short [36]. On the other hand, WHtR has been reported to outperform WC and BMI in relation to the prediction of metabolic syndrome and cardiovascular risk by reflecting height, particularly in Asians [37,38]. Therefore, WHtR is considered an alternative anthropometric marker for visceral obesity [39]. However, other studies have shown that WHtR has similar performance to BMI or WC in predicting cardiovascular risk [40,41]. In short, for the reasons mentioned above, we cannot conclude that one obesity index is better than others. This is consistent with recent studies combining TyG index and obesity indices, which reported conflicting results for predictive value of TyG-related markers. In addition, Er et al reported that, TyG-BMI was stronger predictor of IR than TyG-WC in a recent study involving 511 individuals [19]. On the other hand, Zheng et al., found that TyG-WC was the best marker for the detection of prediabetes and diabetes [20]. Therefore, the superiority of obesity indices remains controversial, and further additional studies on TyG related markers are required.

HOMA-IR is widely used for the quantification of IR, but its cutoffs are inconsistent between studies [42]. The cutoff used for IR in this study was the 75th percentile in men and women from the study population, which resulted in 2,662 men and 2,514 women respectively, and the cutoffs for men and women are generally higher than in other studies [23,24,42]. Therefore, whether our findings can be applied to the general population or to other races should be investigated through further studies.

To our knowledge, this is the first study to assess TyG-WHtR in addition to TyG-BMI and TyG-WC as markers of IR across a large number of participants. However, our study has several limitations. First, because of its cross-sectional design, the associations identified are not prospective, and causality cannot be determined. Further longitudinal studies are necessary to confirm whether TyG-BMI can predict the future occurrence of IR. Second, because the study sample consists primarily of Koreans, the results cannot be generalized to other ethnicities. Given the variability of TG levels according to ethnicity, further research is required to evaluate TyG-BMI, TyG-WC, and TyG-WHtR as general predictors of IR. Considering that recent studies on TyG-BMI have been extended to nonalcoholic fatty liver disease [43], subclinical atherosclerosis [44], and coronary artery disease [45], TyG-related markers deserve further attention with additional studies to identify their associations with risk of cardiovascular disease.

In conclusion, TyG-BMI is a valuable marker to predict IR in healthy Koreans in a minimally invasive and inexpensive manner. It can be easily calculated because required values can be obtained from routine laboratory tests. As such, we recommend the application of TyG-BMI in risk assessments for IR in clinical practice and future epidemiologic studies.
